# Understanding the Spectrum of Flood Syndrome: A Case Series

**DOI:** 10.7759/cureus.62059

**Published:** 2024-06-10

**Authors:** Aditya Jayaprakash, Vikas C Kawarat, Vijayalakshmi S.

**Affiliations:** 1 General Surgery, Madras Medical College, Chennai, IND

**Keywords:** cirrhosis, umbilical hernia, ruptured, spontaneous, flood syndrome

## Abstract

Flood syndrome refers to ruptured umbilical hernias in patients with chronic ascites with underlying liver cirrhosis. These ruptures may introduce infection into the abdomen and hence require emergency surgery. However, these patients are at high risk during these procedures owing to coagulopathy, hypotension and electrolyte imbalances. In our series, we describe six patients who presented with varying degrees of severity and were treated with a standardised protocol of primary anatomic repair and drain placement. Furthermore, we assessed the Child-Turcotte-Pugh (CTP) and Model for End-Stage Liver Disease (MELD) scores in these patients and correlated them to postoperative outcomes. This surgical technique has a good outcome in patients whose CTP and MELD scores predict a safe postoperative period.

## Introduction

Umbilical hernias occur in 20% of patients with cirrhotic liver disease and chronic ascites [1]. The increased intra-abdominal pressure from the ascites and fascial defects caused by dilated peri-umbilical veins predispose to this occurrence [2]. Rarely, in long-standing cases, these hernias may rupture, leaking ascitic fluid and sometimes even intra-abdominal contents from the defect [3]. These events are known as Flood syndrome after Dr. Frank Flood who first described a series of this condition in 1961 [4], although it was first described by Johnson in 1901 [5]. There is an increased incidence of electrolyte abnormalities, coagulopathy and hypotension in these patients as a result of chronic liver disease which greatly increases the risk of surgery [6]. Yet, taking into account the considerable risk of bacterial peritonitis, these patients are candidates for emergency surgery [7]. In our case series, we present six patients with ruptured umbilical hernias presenting with various degrees of severity and progressing to have different outcomes. We preoperatively assessed the Child-Turcotte-Pugh (CTP) score and Model for End-Stage Liver Disease (MELD) score in these patients. All patients were treated with a standardised surgical protocol. We correlate these preoperative scores with the postoperative outcome and show the effectiveness of primary anatomic closure in the treatment of these patients.

## Materials and methods

**Figure 1:**
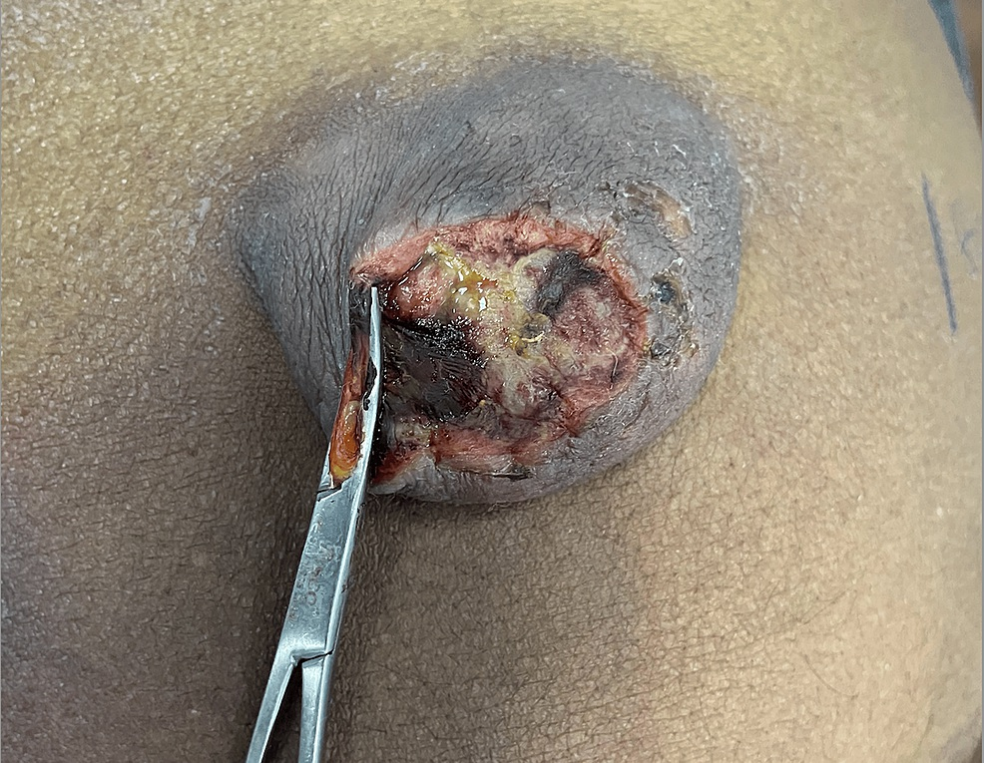
Ruptured umbilical hernia with a sterile clamp applied as a temporary measure

Six consecutive patients who presented to our emergency department over the last two years with Flood syndrome were included in this series. Five of these patients had cirrhotic disease of the liver due to alcoholic liver disease and one due to Hepatitis B. This meant that they all suffered from chronic ascites with an average duration of 11.8 months. They were on medical management for the ascites along with regular abdominal paracentesis. None of the patients suffered from any other comorbid diseases. All patients presented to the emergency department within 24 hours of the hernia rupture. Of the six, five patients had classical Flood syndrome with draining of ascitic fluid alone and one person had evisceration of omentum from the defect. On examination, the patients were icteric with abdomen distention and cachexic peripheries. The umbilical region revealed a deflated hernia with a region of skin necrosis with a defect at its centre, in all patients. A variety of bedside procedures including suturing, sterile dressing, placement of a stoma bag and sterile clamping were utilised for temporary closure of the defect (Figure 1).

**Figure 2:**
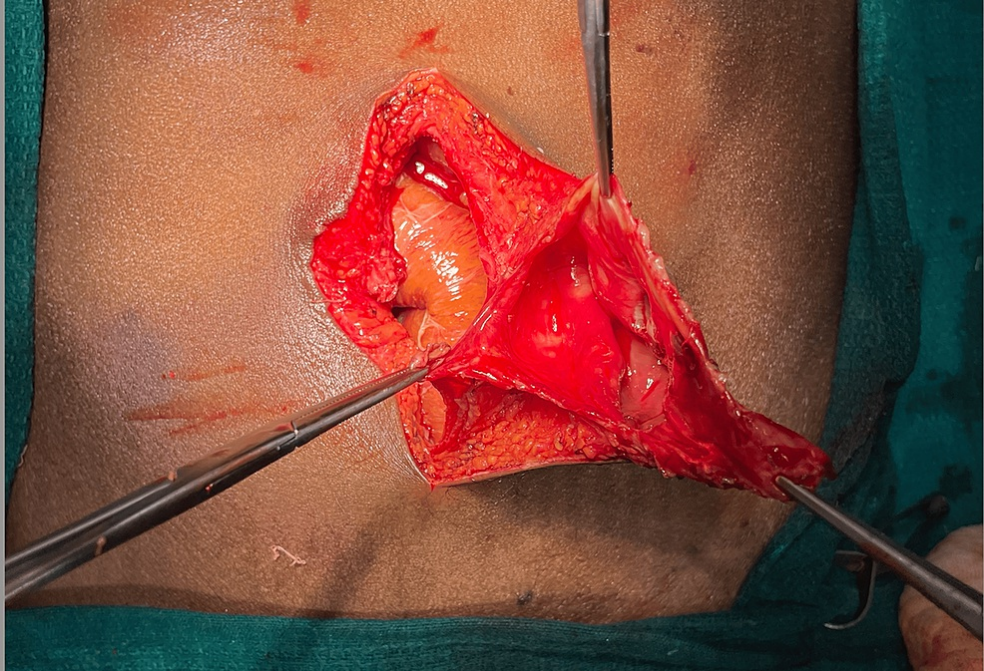
Intraoperative image demonstrating hernial defect and sac

In all patients except the one with evisceration, abdominal CT revealed a fluid-filled sac with a narrow umbilical defect and moderate to severe ascites (Figure 2). Some patients had markedly reduced ascites after rupture and drainage of fluid. Laboratory investigations revealed metabolic derangements including hyponatremia, hypoalbuminemia and coagulopathy. The CTP and MELD scores were calculated on admission. After adequate preoperative optimisation, these patients were taken up for surgery as an emergency procedure. A standardised protocol was followed in all patients which included primary anatomic repair with non-absorbable sutures without mesh placement along with placement of intra-abdominal as well as subcutaneous drain tubes.

Postoperative challenges included further coagulopathy, electrolyte imbalances, hypoalbuminemia and hypotension. The intra-abdominal drains were clamped and carefully released intermittently, taking care to avoid more than 1000mL of ascitic fluid loss per day. Two of the six patients in this study died due to septic shock and coronary artery disease, respectively. Mortality correlated with higher CTP and MELD scores. In the cohort that survived, patients were ambulated early and started on early oral feeds. Oral diuretics and other drugs for the medical management of cirrhosis were resumed as soon as oral feeds were tolerated. The drain tubes were removed as output decreased and the patients were discharged, an average of eight days after surgery. These patients were followed up for a month after surgery and found to have returned to regular activities.

## Results

The predominant metabolic derangements in these patients were hyponatremia (127.8±8.78) and hypoalbuminemia (2.71±0.52). The coagulation profile was abnormal (international normalized ratio (INR): 1.88±0.28), requiring fresh frozen plasma transfusion before surgery in four of the six patients. Table 1 encapsulates the laboratory results of the six patients in our study.

**Table 1:** Laboratory profile of the patients in our series INR: international normalized ratio: NA: sodium, K: potassium: Mod: moderate ascites; Mas: massive ascites

Name	Age/Sex	INR	Urea (mg/dL)	Creatinine (mg/dL)	Total Bilirubin (mg/dL)	Albumin (g/dL)	NA (mmol/L)	K (mmol/L)	Outcome	Ascites
Patient 1	55/M	1.69	21	0.6	1.4	3.0	140	4.1	Survived	Mod
Patient 2	47/M	2.33	26	0.6	14.3	2.8	109	4.0	Died	Mod
Patient 3	45/M	2.3	30	2.7	6.3	2.5	125	3.8	Died	Mod
Patient 4	42/F	1.56	15	0.7	1.6	3.8	137	3.4	Survived	Mas
Patient 5	39/M	1.52	22	0.7	3.4	2.3	126	4.3	Survived	Mod
Patient 6	54/M	1.91	17	0.9	3.0	1.9	130	4.1	Survived	Mas

Of the six patients in our series, two did not survive, bringing the mortality rate to 33%. These patients had a higher CTP score (12-Class C) and MELD score (33.5). Those that survived had an average CTP of 8.75 (Class B) and MELD of 18.75 as shown in Table 2. The patients who died required mechanical ventilation and vasopressor support. In the cohort that survived, there were no surgical site infections or recurrences during the follow-up period.

**Table 2:** CTP and MELD scores and other factors correlated to outcome CTP: Child-Turcotte-Pugh score; MELD: Model for End-Stage Liver Disease score

Name	Age/sex	CTP	MELD	Comorbidities	Length of ICU Stay	Need for Vaso-Pressers	Mechanical Ventilatory Support	Outcome
Patient 1	55/M	7	14	None	Nil	No	No	Survived
Patient 2	47/M	12	32	None	6 days	Yes	Yes	Died
Patient 3	45/M	12	35	None	4 days	Yes	Yes	Died
Patient 4	42/F	7	13	None	Nil	No	No	Survived
Patient 5	39/M	10	25	None	Nil	No	No	Survived
Patient 6	54/M	11	23	None	Nil	No	No	Survived

## Discussion

Flood syndrome was first described by Johnson in 1901, yet it is an eponym of Dr. Frank B. Flood who published the first series of these cases in 1961 [4,5]. Chronic intractable ascites from an underlying cirrhotic liver disease predispose to the formation of umbilical hernias. It is estimated that up to 20% of chronic liver disease patients develop umbilical hernia. The relatively higher pressure of the intra-abdominal compartment due to tense ascites coupled with fascial defects at the peri-umbilical region secondary to dilated veins and superadded nutritional deficiency is believed to contribute to its formation. These hernias are classically ascitic fluid-filled sacs. Chronic high pressure within the sac leads to a zone of skin necrosis that eventually ruptures following sudden increases in intra-abdominal pressure such as coughing or straining. The rupture site is a nidus for peritonitis. Furthermore, these patients are prone to develop electrolyte imbalances as a consequence of their chronic liver disease [6].

Generally, a CT scan of the abdomen is the only preferable radiological investigation before surgery. Various surgical approaches have been described including conservative as well as surgical management. Literature suggests that supportive management alone had a mortality of 60-80% while the surgical approach had a mortality of just 6-20% [7]. Most of the current literature shows that emergency surgery with primary closure is the preferable mode of treatment, as a precaution against the development of bacterial peritonitis [7,8]. The risk of bacterial peritonitis is 18% to 23% as estimated by the literature [1,7]. Novel techniques such as using fibrin glue have been described with good efficacy but inadequate data on recurrence [9]. Recent research has also evaluated the use of transjugular intrahepatic portosystemic shunts (TIPS) without surgery in the management of Flood syndrome to some avail [10]. The literature validates our standard technique in this series [11,12].

The CTP and MELD scores have been validated by the literature as a prognostic tool to evaluate cirrhotic patients undergoing both hepatic as well as non-hepatic surgery [13-17]. Our series suggests that CTP and MELD scores can be used as a guide to predict postoperative mortality in Flood syndrome. Patients with low CTP and MELD scores are candidates for primary surgical repair. In patients with high CTP (Class C) and MELD (>30) scores, the need for surgery must be weighed against the increased peri-operative risk.

This study is limited by a small sample size, lack of randomization and a relatively short period of follow-up. The relative rarity of the diagnosis means that a longer-term systematic study is required to arrive at more definitive conclusions. Furthermore, the hepatic dysfunction confounds the surgical treatment and needs careful matching to obviate any biases.

## Conclusions

Spontaneous rupture of umbilical hernia is a rare occurrence in patients with cirrhotic liver disease. The background of liver disease poses unique challenges to the management of these patients and requires careful perioperative optimisation. The CTP and MELD scores are useful for assessing and managing these cases. These scores are indicators of the degree of metabolic derangement and therefore predict the risk of peri-operative morbidity and mortality. A surgical approach with primary anatomic repair and double drain placement in the emergency setting is suggested as a safe and effective approach to the treatment of Flood syndrome in patients with low CTP and MELD scores. In patients with high CTP (Class C) and MELD (>30) scores, the perioperative risk must be weighed against the benefit of surgery.
